# Ketone Number and Substitution Effect of Benzophenone Derivatives on the Free Radical Photopolymerization of Visible-Light Type-II Photoinitiators

**DOI:** 10.3390/polym13111801

**Published:** 2021-05-29

**Authors:** Tung-Liang Huang, Yung-Chung Chen

**Affiliations:** 1 Department of Chemical and Materials Engineering, National Kaohsiung University of Science and Technology, Kaohsiung 80778, Taiwan; michael915124@gmail.com; 2 Photo-SMART (Photo-Sensitive Material Advanced Research and Technology Center), National Kaohsiung University of Science and Technology, Kaohsiung 80778, Taiwan

**Keywords:** arylamine, benzophenone derivatives, free-radical photopolymerization, mono or diketone units, para-or meta-positions, visible light

## Abstract

Three novel visible-light absorbing benzophenone-based hydrogen acceptors (BPD-D, BPDM-D and BPDP-D) were designed on the basis of a donor–benzophenone–donor structural backbone. Mono or diketone units and double diphenylamine electron-donating groups in para-or meta-positions were introduced to comprehend the electronic and structural effects on free radical photopolymerization (FRPP). Such a structural change leads not only to a red-shift of the absorption maxima but strongly enhances their molar extinction coefficients compared to the commercial phototinitiators such as benzophenone (BP) and 4,4′-bis(diethylamino) benzophenone (EMK). In addition, excellent melting points and thermal decomposition temperatures were achieved for those novel compounds. Further, the photochemical reaction behavior was studied by cyclic voltammograms (CV), photolysis and electron spin resonance (ESR) spectroscopy. Finally, benzophenone derivatives in combination with an amine (TEA, triethylamine) as a co-initiator were prepared and initiated the FRPP of trimethylolpropane trimethacrylate (TMPTMA) using a UV lamp as a light source. When used in stoichiometric amounts, the BPDP-D/TEA had the best double bond conversion efficiency among all the compounds studied, and were even superior to the reference compounds of BP/TEA and EMK/TEA. The results and conclusions could provide the fundamental rules applicable for the structural design of benzophenone derivative-based photoinitiators.

## 1. Introduction

Light-induced radical polymerization reactions have gained attention over traditional polymerization methods in terms of higher polymerization reactivity at ambient temperatures, less energy consumption, solvent-free formulations and lower cost [1,2,3,4,5]. These advantages have resulted in the development of many industrial applications such as coatings, adhesives, microelectronics, 3D objects, dental fillings and some other medical applications [6,7,8,9,10,11,12,13]. Among the photoinitiating processes, the free radical polymerization (FRP) is the most popular approach [14]. Further, the FRP is divided into two types (Type I and Type II) depending on the radical generated route. Type I photoinitiating systems (one-component photoinitiator) are aromatic carbonyl compounds that can react under light irradiation within α-cleavage type to produce initiating radicals [15,16,17,18,19]. Type II photoinitiating systems (two-component photoinitiator) require the presence of an additional co-initiator that can react under light irradiation within the production of radicals and electron/proton transfer processes [20,21,22].

Irrespective of the mechanism of photopolymerization, the photoinitiators play a critical factor as they absorb the light and initiate the polymerization reaction. The reactivity of the photoinitiators dominates the efficiency of polymerization. Researchers showed a number of works regarding the design of new photoinitiators. Currently, it has become a trend to replace traditional ultraviolet (UV) light sources with lower energy consumption (LED light) and/or longer wavelength (visible and near-infrared) exposure light sources [23,24]. This is because of the environmental friendliness, and greater depth of penetration of the latter exposure instrument.

Connected chromophores or conjugation species with photoinitiator unit through covalent bonds are able to efficiently absorb lower energy wavelengths for efficient initiation of photopolymerization. However, initiation of photopolymerization is still a challenge and require more suitable photoinitiators to meet the needs of industry. Previously, we showed that a series of type II benzophenone derivative based photoinitiators containing various mono-electron donating groups in their molecule structures could undergo different photoreactivity under similar light irradiation [25]. There are current reports of the synthesis and characterization of a series of benzophenone derivatives with mono or diketone units at para-or meta-positions with similar diphenylamine electron-donating groups. By comparative studies of these photoinitiators with the commercial analogues, such as benzophenone (BP) and 4,4′-bis(diethylamino) benzophenone (EMK), we confirm their photochemical properties through cyclic voltammetry (CV), photolysis and electron spin resonance (ESR) spectroscopy experiments. In addition, the photoreactivity of all packages were studied via photo differential scanning calorimetry (Photo-DSC).

## 2. Experimental

### 2.1. Materials

Bromobenzene, 4,4′-dibromobenzophenone, diphenylamine, isophthaloyl chloride, terephthaloyl chloride, triethylamine (TEA), trimethylolpropane trimethacrylate (TMPTMA), N-tert-Butyl-α-phenylnitrone (PBN) and all other chemical reagents were purchased from Alfa (Massachusetts, USA) or Sigma-Aldrich (St. Louis, MO, USA). Unless otherwise specified, all of them were used as received. All the solvents were purified by conventional procedures.

### 2.2. Synthesis

#### 2.2.1. BPD-D

A mixture of 4-4′-dibromobenzophenone (1.493 g, 4.400 mmol), diphenylamine (1.486 g, 8.78 mmol), tris(dibenzylideneacetone)dipalladium [Pd_2_(dba)_3_] (0.081 g, 0.088 mmol), sodium t-butoxide (NaOtBu) (1.266 g, 13.17 mmol), and tri-t-butylphosphine P(t-Bu)_3_ (0.351 mL, 0.49 M) in dry toluene (15 mL) was heated at 95 °C overnight in a 100 mL flask under a nitrogen atmosphere. After completion of reaction and cooling, the solvent was removed and the residual mixture was extracted with CH_2_Cl_2_/H_2_O. The collected organic phase was dried using MgSO_4_ and concentrated. Finally, the residue was purified by column chromatography on silica gel using CH_2_Cl_2_: hexane = 1:1 as the eluent to give a goose yellow powder (Yield: 37%). ^1^H NMR (CDCl_3_, 400 MHz): δ (ppm) = 6.99–7.04 (4H, Ar-H), 7.09–7.18 (12H, Ar-H), 7.28–7.34 (8H, Ar-H), 7.66–7.70 (d, 4H, Ar-H, *J* = 8.8 Hz). FT-Mass (M-H^+^) (m/z) (C_37_H_28_N_2_O) calcd. 517.22744; found 517.22726.

#### 2.2.2. BPDM-D

A mixture of the isophthaloyl chloride (3.27 g, 16.12 mmol), bromobenzene (21.92 mL, 209.41 mmol) and AlCl_3_ (5.15 g, 38.62 mmol) was reacted at room temperature and then heated to 95 °C for 12 h. After completion of reaction, to the cooled solution was added HCl(aq) (1 N) to precipitate the solid compound. Finally, it was filtered, and the filtrate was collected and washed with Na_2_CO_3_(aq) until it was neutral and then dried in an oven (100 °C). The product was recrystallized using toluene to yield a white solid of the dibromo-intermediate of the benzophenone derivative BPDM-D (Yield: 70%). ^1^H NMR (CDCl_3_, 400 MHz): δ (ppm) = 7.62–7.71 (6H, Ar-H), 7.98–8.01 (4H, Ar-H), 8.12–8.14 (2H, Ar-H). Subsequently, a mixture of the intermediate (1.266 g, 2.700 mmol), diphenylamine (1.212 g, 7.162 mmol), Pd_2_(dba)_3_ (0.026 g, 0.028 mmol), NaOtBu (0.8384 g, 8.58 mmol) and P(tBu)_3_ (0.176 mL, 0.49 M) in dry toluene (15 mL) was heated at 95 °C overnight in a 100 mL flask under nitrogen atmosphere. After completion of reaction and cooling, the solvent was removed and the residual mixture was extracted with CH_2_Cl_2_/H_2_O. The organic phase collected was dried using MgSO_4_ and concentrated. Finally, the residue was purified by column chromatography on silica gel using CH_2_Cl_2_: hexane = 3:1 as the eluent to give a goose yellow powder (Yield: 62%). ^1^H-NMR (CDCl_3_, 400 MHz): δ (ppm) = 6.98–7.02 (4H, Ar-H), 7.12–7.20 (12H, Ar-H), 7.30–7.35 (8H, Ar-H), 7.54–7.59 (1H, Ar-H), 7.68–7.72 (4H, Ar-H), 7.91–7.95, (2H, Ar-H), 8.10–8.12 (1H, Ar-H). EI-Mass (m/z) (C_44_H_32_N_2_O_2_) calcd. 620.24583; found 620.24555.

#### 2.2.3. BPDP-D

A mixture of terephthaloyl chloride (2.55 g, 12.58 mmol), bromobenzene (7.90 mL, 75.47 mmol) and AlCl_3_ (4.2 g, 31.50 mmol) was reacted at room temperature and then heated at 95 °C for 12 h. After complete reaction, the cooled solution was added HCl(aq) (1 N) to the precipitated solid compound. Finally, it was filtered, and the filtrate was collected and washed with Na_2_CO_3_(aq) until it was neutral and then dried in an oven (100 °C). The product was recrystallized using toluene to give the dibromo-intermediate white solid (Yield: 65%). ^1^H NMR (CDCl_3_, 400 MHz): δ (ppm) = 7.64–7.68 (4H, Ar-H), 7.69–7.73 (4H, Ar-H), 7.82–7.88 (4H, Ar-H). Subsequently, a mixture of the intermediate (1.190 g, 2.700 mmol), diphenylamine (1.140 g, 6.75 mmol), Pd_2_(dba)_3_ (0.025 g, 0.027 mmol), NaOtBu (0.7784 g, 8.099 mmol) and P(tBu)_3_ (0.162 mL, 0.49 M) in dry toluene (15 mL) was heated at 95 °C overnight in a 100 mL flask under nitrogen atmosphere. After completion of reaction and cooling, the solvent was removed and the residual mixture was extracted with CH_2_Cl_2_/H_2_O. The organic phase collected was dried using MgSO_4_ and concentrated. Finally, the residue was purified by column chromatography on silica gel using ethyl acetate: hexane = 1:7 as the eluent to yield a goose yellow powder (Yield: 54%). ^1^H NMR (CDCl_3_, 400 MHz): δ (ppm) = 6.98–7.03 (4H, Ar-H), 7.13–7.20 (12H, Ar-H), 7.31–7.36 (8H, Ar-H), 7.69–7.73 (4H, Ar-H), 7.82 (4H, Ar-H). FT-Mass (M-Na^+^) (m/z) (C_44_H_32_N_2_O_2_) calcd. 643.23560; found 643.23558.

### 2.3. Measurement

^1^H NMR spectra were recorded via an Agilent Unity plus-400 spectrometer (Santa Clara, CA, USA) under room temperature by using CDCl_3_ as d-solvents. Fourier-transfer mass spectrometry (FT-MS) or electron impact mass spectrometry (EI-MS) were recorded using a Bruker APEX II mass spectrometer (Billerica, MA, USA) or a JEOL AccuTOF GCx-plus instrument (Tokyo, Japan), respectively. Ultraviolet–visible (UV-vis) spectra were recorded using a PerkinElmer Lambda 35 UV/VIS Spectrometer (Waltham, MA, USA) in CH_2_Cl_2_ solution (Conc. = 1 × 10^−5^ M). Fluorescence (PL) spectra were recorded using a Hitachi F-4500 Spectrometer (Tokyo, JAPAN) with an excitation wavelength of 360 nm for all compounds in CH_2_Cl_2_ solution (Conc. = 1 × 10^−5^ M). The cyclic voltammetry (CV) was measured using a BioLogic SP-150 model (Seyssinet-Pariset, France) at a scan rate of 100 mV s^−1^ from 0 to −2 V to get the reduction potential for all the benzophenone derivatives. All the measurements were carried out at room temperature in CH_2_Cl_2_ solution (Conc. = 1 × 10^−3^ M) with a conventional three electrode configuration consisting of a platinum working electrode, an auxiliary electrodes, and Ag/Ag^+^ as a reference electrode. Thermogravimetric analysis (TGA) was measured using a TA Instruments SDT Q600 Simultaneous DTA-TGA (New Castle, DE, USA) under nitrogen atmosphere at a heating rate of 15 °C min^−1^ with a sample weight of 3–5 mg. Degradation temperature (T_d_) was taken as the temperature at which 5% weight loss has occurred. Differential scanning calorimetry (DSC) was conducted using a Perkin Elmer DSC 6000 (Waltham, MA, USA) under nitrogen atmosphere at a heating rate of 10 °C min^−1^. Melting point (T_m_) was deduced from the first round of the heating scan.

### 2.4. Photolysis

The changes of the UV–visible absorption intensities based on different phototinitiators were studied using a PerkinElmer Lambda 35 UV/VIS spectrometer (Waltham, MA, USA) under UVP Blak-Ray B-100A UV Lamps (Waltham, MA, USA) (λ: 365 nm, intensity: 100 W) at various exposure times. The PI packages contained BPs (2 × 10^−5^ M) in the presence of TEA (4 × 10^−5^ M) in CH_2_Cl_2_ as the solvent.

### 2.5. ESR Measurement

Electron spin resonance (ESR) experiments on the photoinitiator were carried out using a Bruker EMX Plus X-Band spectrometer (Billerica, MA, USA) using ultrahigh pressure mercury (MUV-250U-L, λ: 250–450 nm, intensity: 250 W) as irradiation source for generating radicals. The radicals were trapped by N-tert-Butyl-α-phenylnitrone (PBN) according to a procedure described according to the literature [25]. All the experiments were carried out at room temperature under nitrogen atmosphere in t-butylbenzene as solvent. The concentrations of PBN, BPs and TEA were 1 × 10^−2^, 2 × 10^−3^ M and 4 × 10^−3^ M, respectively.

### 2.6. Photo-Differential Scanning Calorimetry (Photo-DSC)

The photopolymerization reaction was conducted using photo-differential scanning calorimetry (photo-DSC) on a Perkin Elmer DSC 6000 (Waltham, MA, USA) at 30 °C under nitrogen atmosphere (flow rate: 20 mL/min). A UV lamp (intensity: 225 mW cm^−2^; λ: 250–450 nm) was used as the light source. The BPs/TEA systems with various compositions were prepared as photoinitiating packages and TMPTMA is used as monomer. All of them were mixed together without additional solvent. Mixtures of samples (15 mg) were loaded in an Al pan and irradiation with similar light irradiation. Heat flow versus time curves were obtained during light irradiated process. With integration the area under the exothermic peak, the double bond conversion efficiency (DC) (%) could be determined and calculated by the following Equation (1) [26]:

Double bond conversion efficiency (DC, %) = (ΔH_t_/ΔH_o_^the^°^r^)*100% (1)

where ΔH_t_ is the total reaction heat enthalpy within the exposure time, and ΔH_o_^the^°^r^ is the theoretical reaction heat enthalpy for the complete conversion of acrylic monomer. The ΔH_o_^the^°^r^ was 86 KJ/mol [27]. Further, the rate of polymerization (Rp) is directly related to the heat flow (dH/dt) by the Equation (2):

Rate of polymerization (Rp) = dH/dt(2)

## 3. Results and Discussion

### 3.1. Synthesis and Basic Properties

**Scheme 1:**
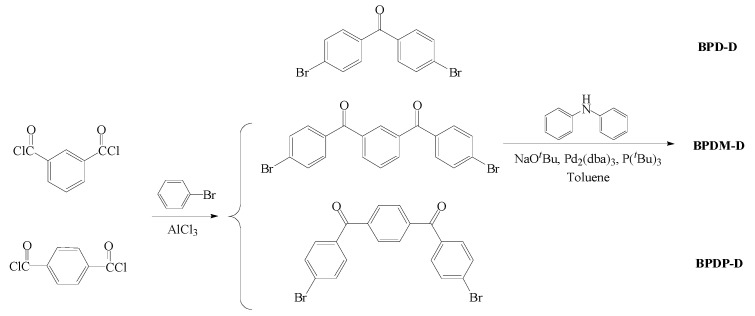
Synthesis route of double donors-based benzophenone derivatives (BPD-D, BPDM-D and BPDP-D).

**Figure 1:**
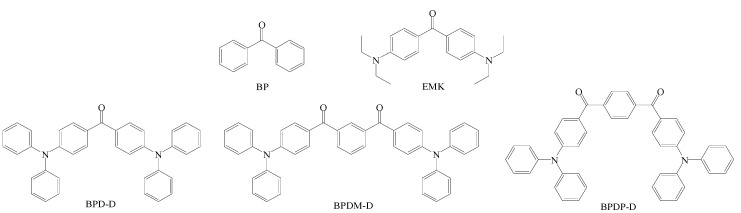
Structures of benzophenone (BP), 4,4′-Bis(diethylamino) benzophenone (EMK) and double donors-based benzophenone derivatives (BPD-D, BPDM-D and BPDP-D).

The synthesis of novel type II benzophenone derivatives were carried out using a facile two step reaction that including (a) Buchwald–Hartwig C-N-coupling reaction between various dibromo compounds and diphenylamine; (b) Friedel–Crafts reaction between isophthaloxy chloride and bromobenzene. The synthetic routes of the three target compounds (BPD-D, BPDM-D and BPDP-D) are illustrated in Scheme 1. The corresponding structures are also shown in Figure 1. In particular, these three benzophenone derivatives can be used to study the effect of the number of ketone groups and located at meta/para positions. We also used commercial photoinitiators, BP (benzophenone) and EMK (4,4′-bis(diethylamino)-benzophenone) (Figure 1) for further comparison. All of the three novel photoinitiators—namely, BPD-D, BPDM-D and BPDP-D—displayed goose yellow powder appearance after purification through chromatography on silica gel. The compounds were characterized through by ^1^H NMR (Figures S1–S3) and mass spectrometry (Figures S4–S6). In the case of each of the compounds, the mass spectra showed agreement with the calculated values. The ^1^H NMR spectra of these compounds are consistent with the proposed structures, showing the expected features with correct integration proton areas.

Solubility is an important property for a photoinitiator. Therefore, the solubility of all the new compounds and the reference compounds, namely BP and EMK are tested using various solvents. The corresponding results were listed in Table 1. In addition, we also conducted the solubility with commercial monomers such as TMPTA (trimethylolpropane triacrylate) and TMPTMA (trimethylolpropane trimethacrylate). All the samples showed good solubility in the test solvents (DMF, DCM, THF and acetone). However, the three synthesized samples exhibited poor solubility in MeOH compared to BP and EMK due to the presence of a higher number of benzene rings among the structures of BPD-D, BPDM-D and BPDP-D. Moreover, the new compounds showed poor solubility in TMPTA and a better solubility in TMPTMA. The TMPTMA displayed better solubility than TMPTA due to the presence of bulky methacrylate group. However the TMPTMA showed relative slower photoreactivity than TMPTA [28]. To avoid the aggregation, in this work, we selected TMPTMA as the tested monomer to understand the photoreactivity.

### 3.2. Optical Properties

**Figure 2:**
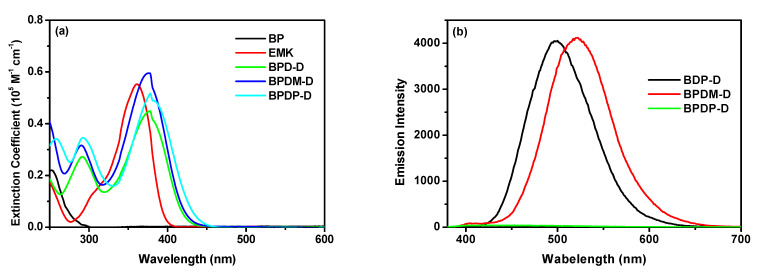
(**a**) UV/Vis and (**b**) photoluminescence (PL) spectra of the photoinitiators in CH_2_Cl_2_ solutions (Conc. = 1 × 10^−5^ M).

The absorption spectra of the reference compounds (BP and EMK) and newly synthesized photoinitiators (BPD-D, BPDM-D and BPDP-D) in dichloromethane were shown in Figure 2a and the corresponding data was summarized in Table 2. Reference BP compound exhibited a major absorption band at around 250 nm and a very weak absorption in the region of 360 nm. On the contrary, the reference EMK compound exhibited a larger absorption band in the region of 360 nm due to the presence of electron-donating diethylamino substituents. In comparison to the reference compounds, all the new compounds displayed two or three major absorption bands in the region of 250–450 nm. The bands at relatively shorter wavelength were attributed to the π–π* transition of the aromatic rings. The bands in the longer wavelength region (~378 nm) were ascribed to the intramolecular charge transfer transition from arylamine donor to ketone acceptor. The λ_max_ values of all the new photoinitiators are similar, but were red-shifted compared to BP and EMK compounds. These results indicate that all the new photoinitiators were more suitable and may be good candidates for FRP under visible light irradiation. BPDP-D exhibited the broadest absorption spectra due to the diketone with *para* electron-donation substituents, which might be beneficial for light absorption with good photoreactivity. In addition, BPD-D sample containing double arylamine substituents was also compared to the mono-arylamine substituent (i.e., BP-1) [25]. The BPD-D has longer λ_max_ and higher molar extinction coefficient than that of BP-1 congener.

Figure 2b shows the PL emission spectra of the new compounds with the excitation wavelength at 360 nm. Except BPDP-D, BPD-D and BPDM-D exhibited emission peaks with strong intensity at 500 and 522 nm, respectively. The lower emission intensity of the BPDP-D compound indicating that the singlet state was deactivated via non-radiative transition and could pass through the intersystem to the triplet state [29]. Thus, a higher triplet state concentration may effectively increase the degree of photopolymerization, although contribution from other factors cannot be ruled out.

### 3.3. Electrochemical Properties

**Figure 3:**
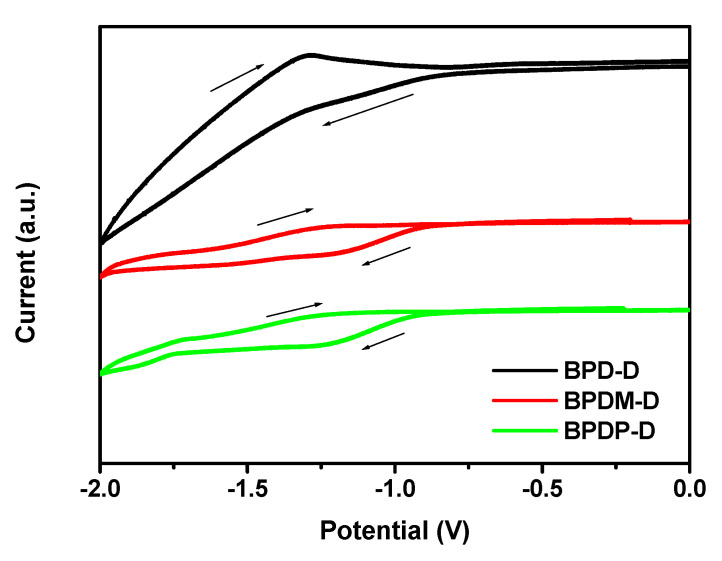
Cyclic voltammograms of the BPD-D, BPDM-D and BPDP-D recorded in CH_2_Cl_2_ solutions (scan rate 100 mVs^−1^).

To investigate the redox property of the photoinitiators, cyclic voltammetry was performed in DCM solution using of 0.1 M tetrabutylammonium hexafluorophosphate (Bu_4_NPF_6_) as the support electrolyte. The corresponding cyclic voltammograms were shown in Figure 3. The results were summarized in Table 2. The reduction potential of the newly synthesized compounds is in the range of −1.04 to −1.08 V. For comparison, the reduction potential of the BP was −1.13 V. All the new photoinitiators exhibited higher reduction potentials due to the presence of electron-donating arylamine groups. We also calculated free energy changes, ΔG_ET_, to evaluate the feasibility of the photoinduced electron transfer (PET) of the bimolecular systems from Type II photoinitiators (benzophenone derivatives) to the selected hydrogen donor, TEA, using the classical Rehm–Weller equation [30]. In the following equation: ΔG_ET_ = E_ox_ − E_red_ − E_g_ + C, E_ox_ is the oxidation potential of the TEA, E_red_ is the reduction potential of the benzophenone derivatives, E_g_ is the band gap of the TEA, and C is the electrostatic interaction energy for the initially formed ion pair, generally considered as negligible in polar solvents [30]. ΔG_ET_ values of all the biomolecular systems are in the range of −3.36 to −3.45 eV. ΔG_ET_ values for the packages are all negative suggesting favorable intermolecular electron transfer processes.

### 3.4. Thermal Properties

**Figure 4:**
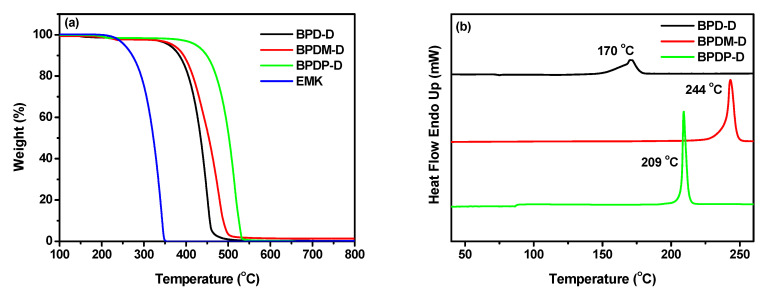
(**a**) TGA thermograms and (**b**) DSC curves of the photoinitiator compounds in nitrogen atmosphere.

The thermal properties, namely, temperature of 5% weight loss (T_d_) and melting point temperature (T_m_) of the photoinitiators were deduced from thermogravimetric analysis (TGA) and differential scanning calorimetry (DSC) at heating rates at 15 and 10 °C min^−1^ under nitrogen atmosphere. The corresponding curves and results were present in Figure 4 and Table 2. The T_d_ values of the benzophenone derivatives BPD-D, BPDM-D and BPDP-D were at 360, 369 and 424 °C, respectively (Figure 4a). Although the structure of these compounds are similar, their T_d_ values varied significantly. For example, photoinitiators BPDM-D and BPDP-D, comprising of two ketone moieties, exhibited relatively higher T_d_ values than the mono-ketone compound (BPD-D). In addition, the *para*-substituted of the BPDP-D showed the highest T_d_ value at 424 °C. The thermal properties of the photoinitiators, namely, BPD-D, BPDM-D and BPDP-D were compared to the reference compounds, BP and EMK. Clearly, the T_d_ values of the new photoinitiators surpassed those of the commercial BP (T_d_ = 115 °C) and EMK (T_d_ = 254 °C) compounds. This indicates that all the novel compounds possess good thermal stability to meet the requirements for photopolymerization process. The melting point (T_m_) values of the compounds obtained from the DSC traces were shown in Figure 4b. The T_m_ values are dependent on the substituted position and the number of ketone units. The T_m_ values increased in the order of BPDM-D (244 °C) > BPDP-D (209 °C) > BPD-D (170 °C). The corresponding values for the reference compounds of BP and EMK were 49 and 97 °C, respectively. Again, all the new compounds showed higher melting points, which indicated that they have wide range of operation temperatures and storage stabilities.

### 3.5. Photolysis and ESR Studies

**Figure 5:**
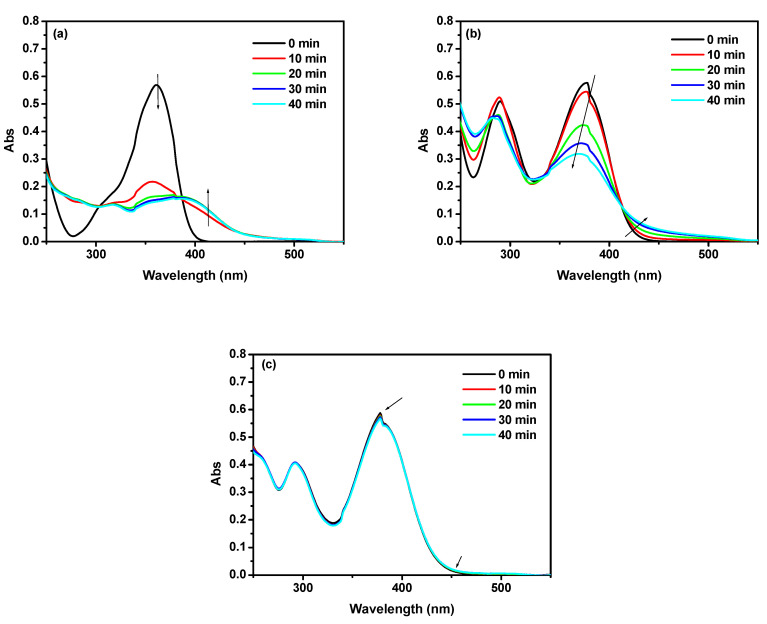
Photolysis of the (**a**) EMK/TEA (2 × 10^−5^ M/4 × 10^−5^ M), (**b**) BPD-D/TEA (2 × 10^−5^ M/4 × 10^−5^ M), and (**c**) BPDP-D/TEA (2 × 10^−5^ M/4 × 10^−5^ M) formulations in CH_2_Cl_2_ solution at various exposure time.

The changes of the UV spectra of selected BPs in the presence of TEA under the same concentration were recorded upon light irradiation for different exposure times. The corresponding photolysis behavior were shown in Figure 5. The absorbance values at 360 nm EMK/TEA and BPD-D/TEA packages obviously decrease, while the absorbance increases below 300 nm and above 400 nm as continuous irradiation. This results indicating the photolysis process taking place. Moreover, the absorption band located at around 360 nm was rapidly blue shifted without isosbestic point [31]. On the other hand, the BPDP-D/TEA package showed insignificant UV spectral change after irradiation. Although the former formulations had faster photoinduced bond dissociation detected by photolysis, the triplet state lifetime and electron transfer efficiency cannot be ruled out.

**Figure 6:**
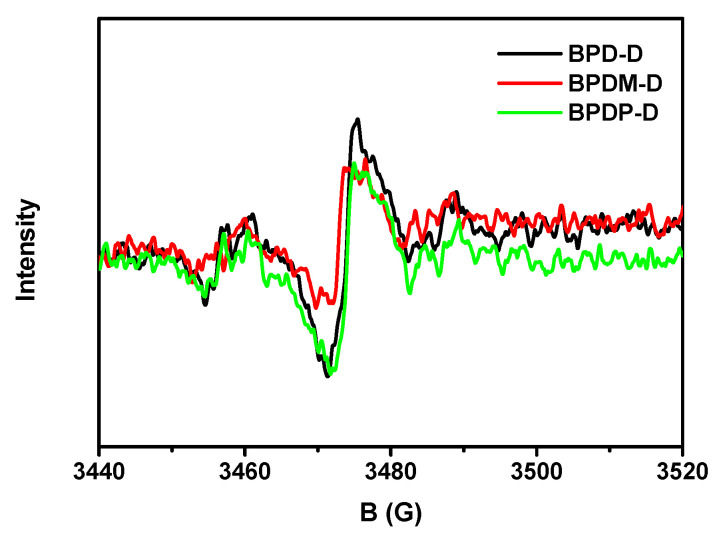
Experimental ESR spectra of the radicals generated by various hydrogen acceptors (BP derivatives) with hydrogen donor (TEA) irradiated upon UV lamp for 6 min and trapped by N-tert-Butyl-α-phenylnitrone (PBN) in *t*-butylbenzene solution.

Figure 6 illustrates the representative ESR spectra by various hydrogen acceptors (BPD-D, BPDM-D and BPDP-D) with hydrogen donor (TEA) irradiated upon UV lamp (wavelength, 250 < λ < 450 nm) at 6 min. All the photoinitiating systems showed similar ESR signals, suggesting the generating mechanism of the active free radicals through these packages should be similar. The radical concentration towards the packages decreases in the order of BPD-D/TEA > BPDP-D/TEA > BPDM-D/TEA. This observation implies that the composition of BPD-D/TEA may have the highest radical concentration, and thus the expected highest photoreactivity.

### 3.6. Photopolymerization

**Figure 7:**
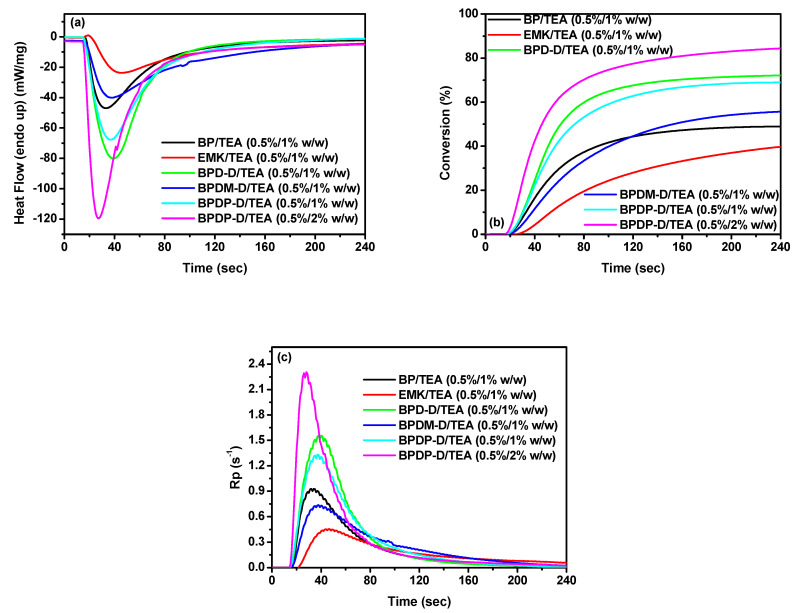
(**a**) Heat flow versus time; (**b**) conversion versus time; (**c**) Rp versus time of TMPTMA photopolymerization initiated by BPs/TEA systems. The irradiation starts for t = 24 s.

We then investigated the photoreactivity of various photoinitiating systems (BPD-D/TEA, BPDM-D/TEA and BPDP-D/TEA) using photo-differential scanning calorimetry (photo-DSC). In addition, the reference formulations (BP/TEA and EMK/TEA) were prepared for comparison. The corresponding results were shown in Figure 7 and Table 3. As the BPs/TEA systems were at a weight ratio of 0.5/1 wt %, the maximum heat flow and the polymerization rate decreased in the order of BPD-D/TEA > BPDP-D/TEA > BP/TEA > BPDM-D/TEA > EMK/TEA and BPD-D/TEA > BPDP-D/TEA > BP/TEA > BPDM-D/TEA > EMK/TEA, respectively. In addition, the final double bond conversion efficiency irradiated at 4 min decreased in the order of BPD-D/TEA (72%) > BPDP-D/TEA (69%) > BPDM-D/TEA (56%) > BP/TEA (49%) > EMK/TEA (42%). Obviously, the DC values of all the new formulations were shown to be superior to that of the reference systems. This might be due to the introduction of arylamine electron-donating groups, which leads to a good light harvesting in the region of 250 to 450 nm. Although EMK also has good light harvesting ability, the amine in its structure can also be used as a hydrogen donor, and competition may occur when the additional hydrogen donor, TEA, is introduced. BPD-D/TEA was compared to the BPDP-D/TEA and BPDM-D/TEA, the former had the highest conversion efficiency as well as the highest heat flow and polymerization rate. This is because the latter had two ketone groups, so the ratio of BPs to TEA was only half compared to the BPD-D/TEA. In addition, the result was consistent with the ESR data mentioned above. Moreover, the BPDM-D/TEA package exhibited lower DC value than BPDP-D/TEA caused the poor solubility of BPDM-D resulting poor dispersion under the tested TMPTMA monomer. We further exam BPDP-D/TEA at a weight ratio of 0.5/2 wt % to study its photoreactivity. As a result, this package showed the maximum heat flow and faster polymerization rate with highest double conversion efficiency (85%). Therefore, BPDP-D/TEA photoinitiating system achieved the best conversion efficiency among all under the equal quantity of ketone number to TEA compound.

## 4. Conclusions

In this study, benzophenone is used as a scaffold for the syntheses of a series of hydrogen acceptors (BPD-D, BPDM-D and BPDP-D) with mono or diketone units and in para-or meta-positions of arylamine electron-donating groups. These novel compounds absorb light in the near UV/visible region at around 380 nm on their maximum absorption peaks. They also exhibited moderately organic solubility and good thermal stabilities. The photochemical mechanism of the photoinitiating systems was studied via photolysis, cycle voltammetry and ESR experiments. Finally, all the new benzophenone derivatives together with the coinitiator (TEA) could efficiently initiate the polymerization of TMPTMA irradiated by a similar light source. Further, the polymerization rate is also superior to the reference samples (BP/TEA and EMK/TEA). Under the equal quantity of weight ratios, the BPDP-D/TEA package appeared to have the best conversion efficiency among them all. We demonstrated that the structural change affecting the photoinitiating properties corresponds to a ketone numbers and substitution effect on the benzophenone building block.

## Figures and Tables

**Table 1:** Solubility of the photoinitiators ^a^.

Sample	DMF	DCM	THF	Acetone	Methanol	TMPTA ^b^	TMPTMA ^c^
BP	○	○	○	○	○	○	○
EMK	○	○	○	○	○	○	○
BPD-D	○	○	○	○	Δ	Δ	○
BPDM-D	○	○	○	○	Δ	X	Δ
BPDP-D	○	○	○	○	Δ	X	○

^a^ Solubility was determined with 1 mg of photoinitiator in 1 mL of solvent. O = soluble at room temperature; Δ = soluble on heating; X = insoluble even on heating. ^b^ TMPTA: trimethylolpropane triacrylate. ^c^ TMPTMA: trimethylolpropane trimethacrylate.

**Table 2:** Electrooptical parameters and thermal properties of the compounds.

Sample	λ_abs_ (ε*10^5^ M ^−1^cm ^−1^) (nm) ^a^	E_ox_ (V) ^b^	E_red_ (V) ^c^	E_g_ (eV) ^d^	ΔG_ET_ (eV) ^e^	T_d_ (°C) ^f^	T_m_ (°C) ^g^
BP	253 (0.220), 363 (0.0251)	--	−1.13 ^h^	--	−3.36	115 ^i^	49 ^i^
EMK	240 (0.1849), 361 (0.5528)	--	--	--	--	254	97 ^i^
BPD-D	292 (0.2722), 378 (0.4492)	--	−1.08	--	−3.41	360	170
BPDM-D	290 (0.3162), 376 (0.5956)	--	−1.04	--	−3.45	369	244
BPDP-D	258 (0.3411), 292 (0.3458), 378 (0.5176)	--	−1.07	--	−3.42	424	209
TEA	--	0.53	--	5.02	--	--	--

^a^ Recorded in DCM solutions at 10^−5^ M. ^b^ Oxidation potential. Recorded in DCM solutions. Scan rate, 100 mV s^−1^; electrolyte, nBu_4_NPF_6_. ^c^ Reduction potential. Recorded in DCM solutions. Scan rate, 100 mV s^−1^; electrolyte, nBu_4_NPF_6_. ^d^ The bandgap, E_g_, was derived from the offset of the absorption spectra. ^e^ From the Rehm–Weller equation. ΔG_ET_ = E_ox_ − E_red_ − E_g_. E_ox_: oxidation potential of TEA; E_red_: reduction potential of HABIs; E_g_: band gap of TEA. ^f^ Temperatures at 5% weight loss recorded by TGA at a heating rate of 15 °C/min in N_2_. ^g^ Melting point. ^h^ The reduction potential were tabulated from the literature [25]. ^i^ Data from materials safety data sheet document.

**Table 3:** Photo-DSC data derived from BPs/TEA packages ^a^.

Sample	Final Conversion (%)	ΔHt (kJ/mol) ^b^	*H_max_* (mW/mg) ^c^	Rp_max_ (s^−1^) ^d^	*T_max_* (s) ^e^
BP/TEA	49	42	47	0.93	33
EMK/TEA	42	36	24	0.45	46
BPD-D/TEA	72	62	80	1.56	39
BPDM-D/TEA	56	48	40	0.73	38
BPDP-D/TEA	69	59	68	1.33	39
BPDP-D/TEA ^f^	85	73	120	2.30	26

^a^ Photo-DSC of the BPs/TEA blend at ratios of 0.5/1 (*w*/*w*), measured with 225 mW cm^−2^ of UV light for 4 min. ^b^ ΔH_t_ is the total reaction heat enthalpy within 4 min. ^c^ H_max_: maximum heat flow values. ^d^ Rp_max_: maximum rate of polymerization. ^e^ T_max_: time at maximum heat flow. ^f^ BPDP-D/TEA at ratio of 0.5/2 (*w*/*w*).

## Data Availability

The data presented in this study are available on request from the corresponding author.

## Supplementary Materials

The following are available online at https://www.mdpi.com/article/10.3390/polym13111801/s1, Figure S1: ^1^H NMR of the BPD-D, Figure S2: ^1^H NMR of the BPDM-D, Figure S3: ^1^H NMR of the BPDP-D, Figure 4: Mass and HR-MS of the BPD-D, Figure S5: EI-MS of the BPDM-D, Figure S6: Mass and HR-MS of the BPDP-D.

## References

[1] Xiao P., Zhang J., Dumur F., Tehfe M.A., Morlet-Savary F., Graff B., Gigmes D., Fouassier J.P., Lalevée J. (2015). Visible light sensitive photoinitiating systems: Recent progress in cationic and radical photopolymerization reactions under soft conditions. Prog. Polym. Sci..

[2] Yilmaz G., Yagci Y. (2020). Light-induced step-growth polymerization. Prog. Polym. Sci..

[3] Malik M.S., Schlögl S., Wolfahrt M., Sangermano M. (2020). Review on UV-Induced Cationic Frontal Polymerization of Epoxy Monomers. Polymers.

[4] Chen M., Zhong M., Johnson J.A. (2016). Light-Controlled Radical Polymerization: Mechanisms, Methods, and Applications. Chem. Rev..

[5] Xiao P., Zhang J., Zhao J., Stenzel M.H. (2017). Light-induced release of molecules from polymers. Prog. Polym. Sci..

[6] Singh M., Rana S., Agarwal S. (2020). Light induced morphological reforms in thin film of advanced nano-materials for energy generation: A review. Opt. Laser Technol..

[7] Corrigan N., Boyer C. (2020). In the Limelight: 2D and 3D Materials via Photo-Controlled Radical Polymerization. Trends Chem..

[8] Samadian H., Maleki H., Allahyari Z., Jaymand M. (2020). Natural polymers-based light-induced hydrogels: Promising biomaterials for biomedical applications. Coord. Chem. Rev..

[9] Kowalska A., Sokolowski J., Bociong K. (2021). The Photoinitiators Used in Resin Based Dental Composite—A Review and Future Perspectives. Polymers.

[10] Tomal W., Ortyl J. (2020). Water-Soluble Photoinitiators in Biomedical Applications. Polymers.

[11] Liu S., Chen H., Zhang Y., Sun K., Xu Y., Morlet-Savary F., Graff B., Noirbent G., Pigot C., Brunel D. (2020). Monocomponent Photoinitiators based on Benzophenone-Carbazole Structure for LED Photoinitiating Systems and Application on 3D Printing. Polymers.

[12] Liu F., Liu A., Tao W., Yang Y. (2020). Preparation of UV curable organic/inorganic hybrid coatings-a review. Prog. Org. Coatings.

[13] Fuchs Y., Soppera O., Haupt K. (2012). Photopolymerization and photostructuring of molecularly imprinted polymers for sensor applications—A review. Anal. Chim. Acta.

[14] Xiao P., Lalevée J., Allonas X., Fouassier J., Ley C., El-Roz M., Shi S.Q., Nie J. (2010). Photoinitiation mechanism of free radical photopolymerization in the presence of cyclic acetals and related compounds. J. Polym. Sci. Part. A Polym. Chem..

[15] Dietlin C., Trinh T.T., Schweizer S., Graff B., Morlet-Savary F., Noirot P.-A., Lalevée J. (2020). New Phosphine Oxides as High Performance Near-UV Type I Photoinitiators of Radical Polymerization. Molecules.

[16] Breloy L., Negrell C., Mora A.-S., Li W.S.J., Brezová V., Caillol S., Versace D.-L. (2020). Vanillin derivative as performing type I photoinitiator. Eur. Polym. J..

[17] You J., Cao D., Hu T., Ye Y., Jia X., Li H., Hu X., Dong Y., Ma Y., Wang T. (2021). Novel Norrish type I flavonoid photoinitiator for safe LED light with high activity and low toxicity by inhibiting the ESIPT process. Dye. Pigment..

[18] Liao W., Xu C., Wu X., Liao Q.Y., Xiong Y., Li Z., Tang H.D. (2020). Photobleachable cinnamoyl dyes for radical visible pho-toinitiators. Dye. Pigment..

[19] Qiu W., Li M., Yang Y., Li Z., Dietliker K.P. (2020). Cleavable coumarin-based oxime esters with terminal heterocyclic moieties: Photobleachable initiators for deep photocuring under visible LED light irradiation. Polym. Chem..

[20] Li Y.-H., Chen Y.-C. (2020). Triphenylamine-hexaarylbiimidazole derivatives as hydrogen-acceptor photoinitiators for free radical photopolymerization under UV and LED light. Polym. Chem..

[21] Chen Y.-C., Liu T.-Y., Li Y.-H. (2021). Photoreactivity study of photoinitiated free radical polymerization using Type II photoinitiator containing thioxanthone initiator as a hydrogen acceptor and various amine-type co-initiators as hydrogen donors. J. Coatings Technol. Res..

[22] Tang L., Nie J., Zhu X. (2020). A high performance phenyl-free LED photoinitiator for cationic or hybrid photopolymerization and its application in LED cationic 3D printing. Polym. Chem..

[23] Shao J., Huang Y., Fan Q. (2014). Visible light initiating systems for photopolymerization: Status, development and challenges. Polym. Chem..

[24] Pigot C., Noirbent G., Brunel D., Dumur F. (2020). Recent advances on push–pull organic dyes as visible light photoinitiators of polymerization. Eur. Polym. J..

[25] Huang T.L., Li Y.H., Chen Y.C. (2020). Benzophenone derivatives as novel organosoluble visible light TypeIIphotoinitiators fo-rUVandLEDphotoinitiating systems. J. Polym. Sci..

[26] Jiang X., Yin J. (2004). Dendritic Macrophotoinitiator Containing Thioxanthone and Coinitiator Amine. Macromolecules.

[27] Andrzejewska E., Andrzejewski M. (1998). Polymerization kinetics of photocurable acrylic resins. J. Polym. Sci. Part. A Polym. Chem..

[28] Dworak C., Koch T., Varga F., Liska R. (2010). Photopolymerization of biocompatible phosphorus-containing vinyl esters and vinyl carbamates. J. Polym. Sci. Part. A Polym. Chem..

[29] Hammond G.S., Moore W.M. (1959). The role of a triplet state in the photoreduction of benzophenone. J. Am. Chem. Soc..

[30] Rehm D., Weller A. (1970). Kinetics of Fluorescence Quenching by Electron and H-Atom Transfer. Isr. J. Chem..

[31] Li J., Zhang X., Ali S., Akram M.Y., Nie J., Zhu X. (2019). The effect of polyethylene glycoldiacrylate complexation on type II photoinitiator and promotion for visible light initiation system. J. Photochem. Photobiol. A Chem..

