# Supplementary material for: Ketone Number and Substitution Effect of Benzophenone Derivatives on the Free Radical Photopolymerization of Visible-Light Type-II Photoinitiators
Source: Polymers (Basel). 2021 May 29;13(11):1801. doi: 10.3390/polym13111801 (PMC8199044; doi:10.3390/polym13111801)
Supplement: Supplementary file 1 [file polymers-13-01801-s001.zip › polymers-1236928-SI.pdf]

Supporting Information

Ketone Number and Substitution Effect of Benzophenone Derivatives on the Free Radical Photopolymerization of Visible-light Type-II Photoinitiators

Tung-Liang Huang[1], Yung-Chung Chen[1,2*]

[1.] Department of Chemical and Materials Engineering, National Kaohsiung University of Science and Technology, Kaohsiung 80778, Taiwan, ROC

[2.] Photo-SMART (Photo-sensitive Material Advanced Research and Technology Center), National Kaohsiung University of Science and Technology, Kaohsiung City 80778, Taiwan ROC

*Corresponding authors: Y. C. Chen (chenyc@nkust.edu.tw)

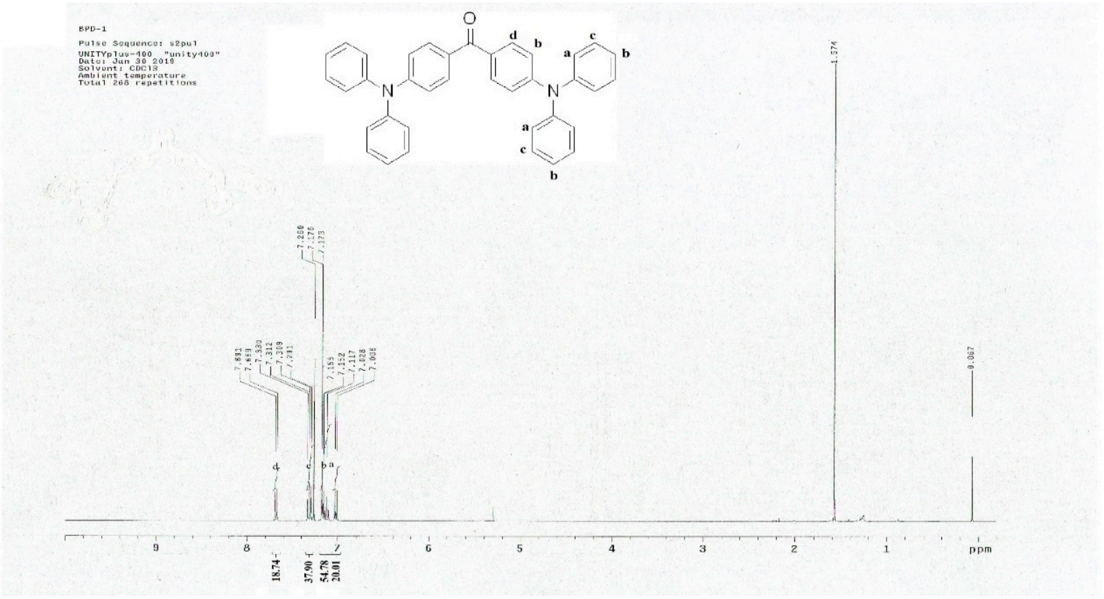

Figure S1. ^{1}H NMR of the **BPD-D**.

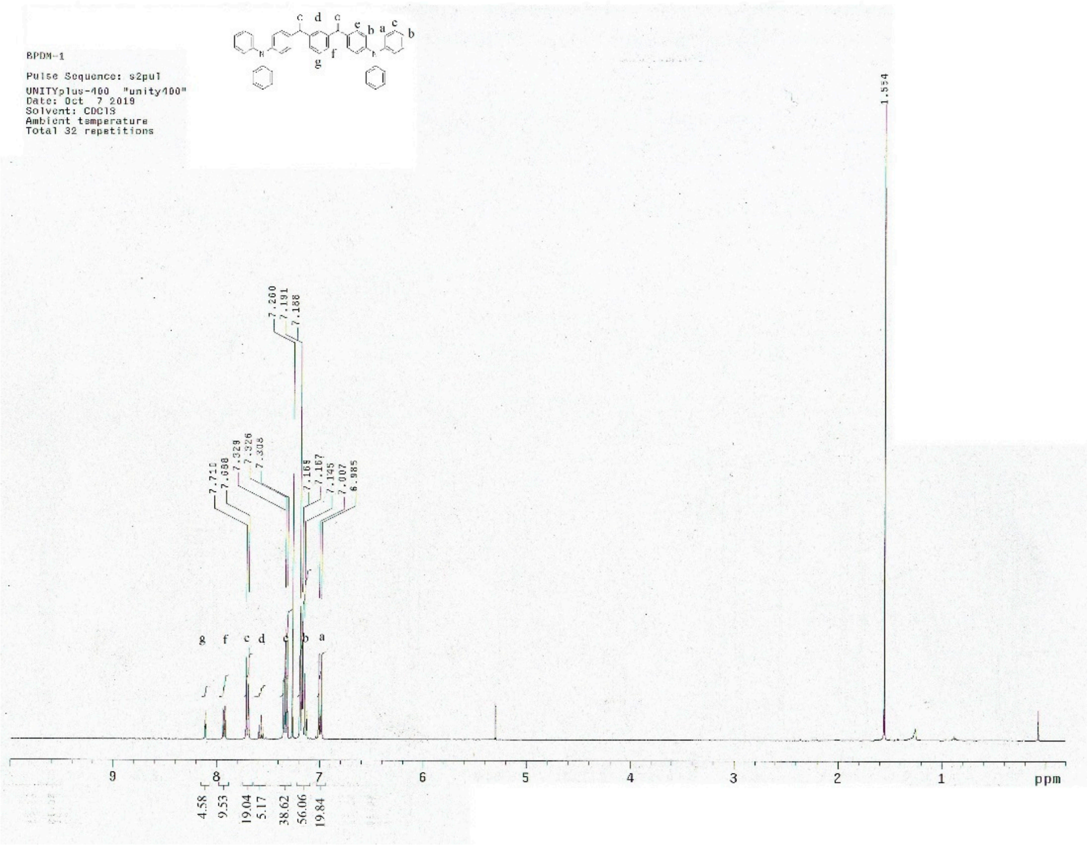

Figure S2. ^{1}H NMR of the **BPDM-D**.

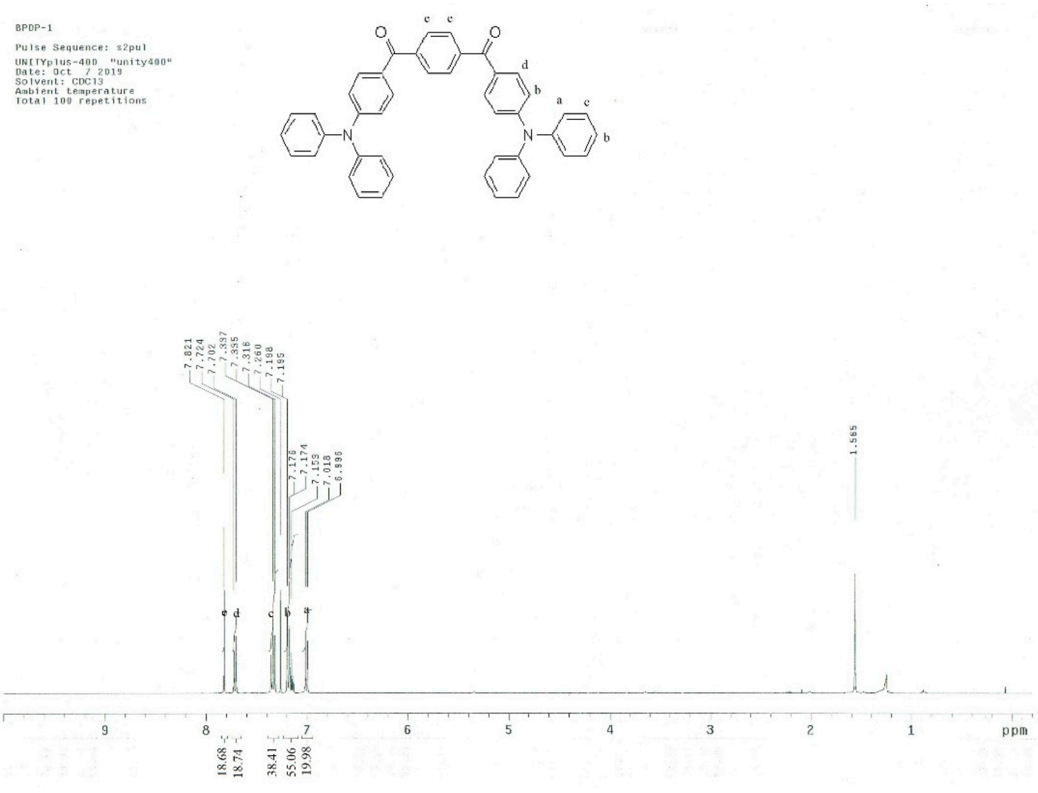

Figure S3. ^{1}H NMR of the **BPDP-D**.

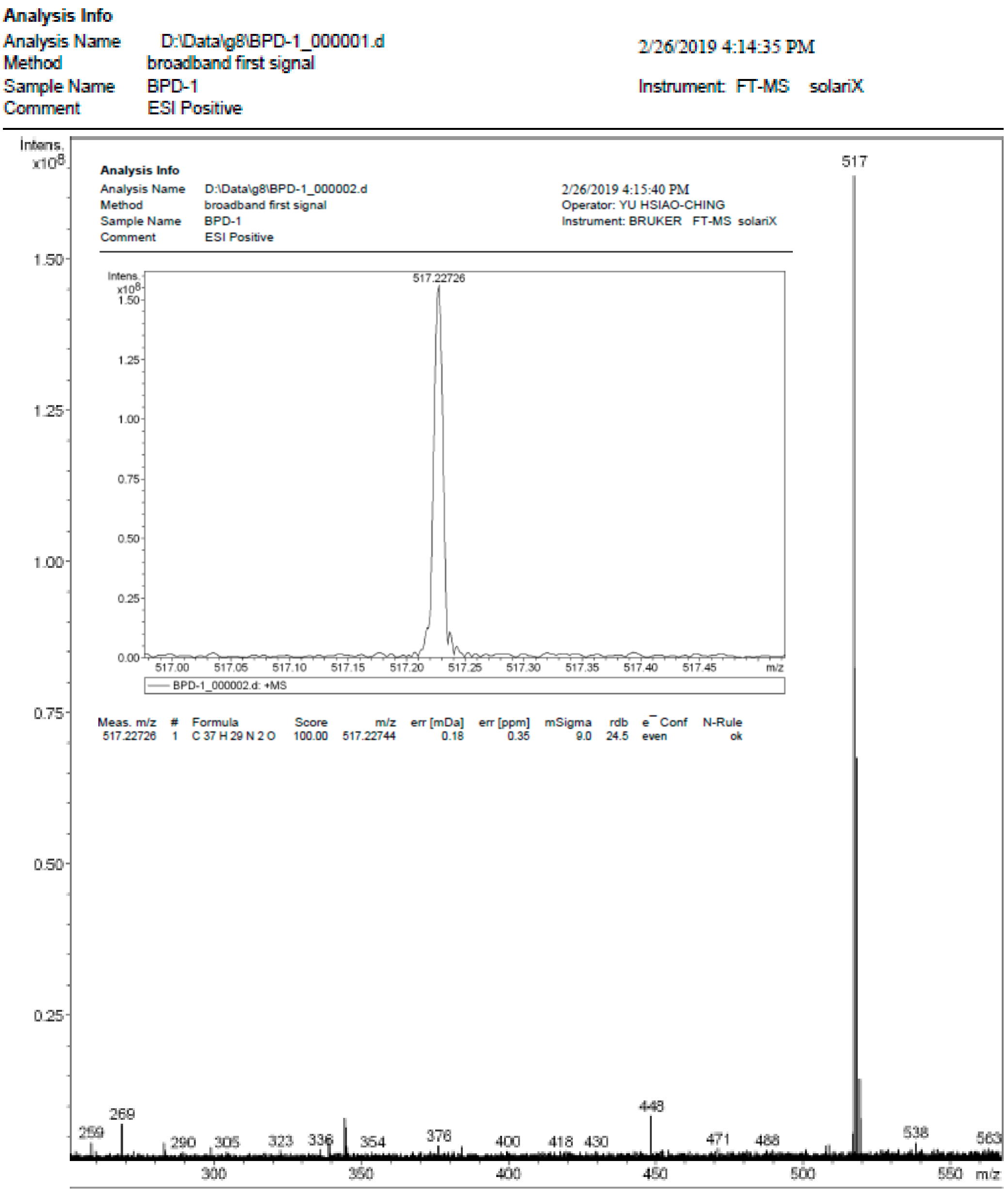

Figure S4. Mass and HR-MS of the **BPD-D**.

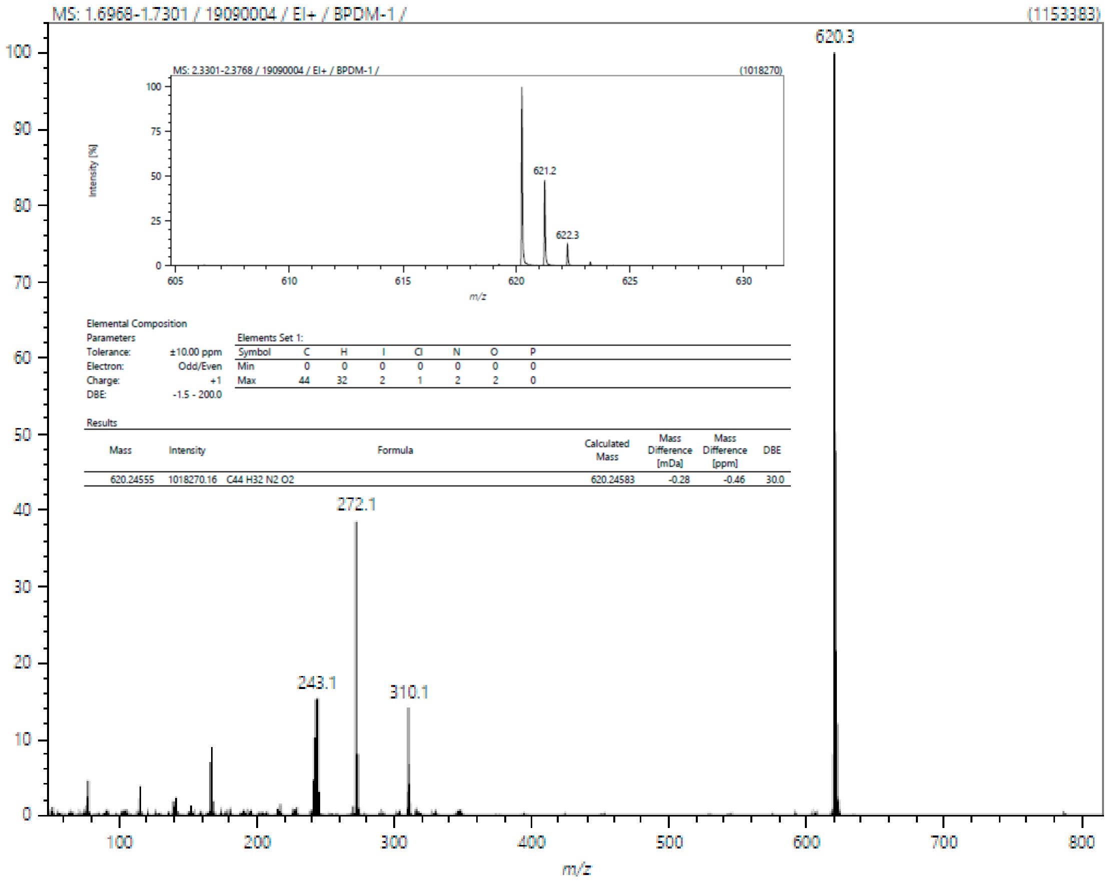

Figure S5. EI-MS of the **BPDM-D**.

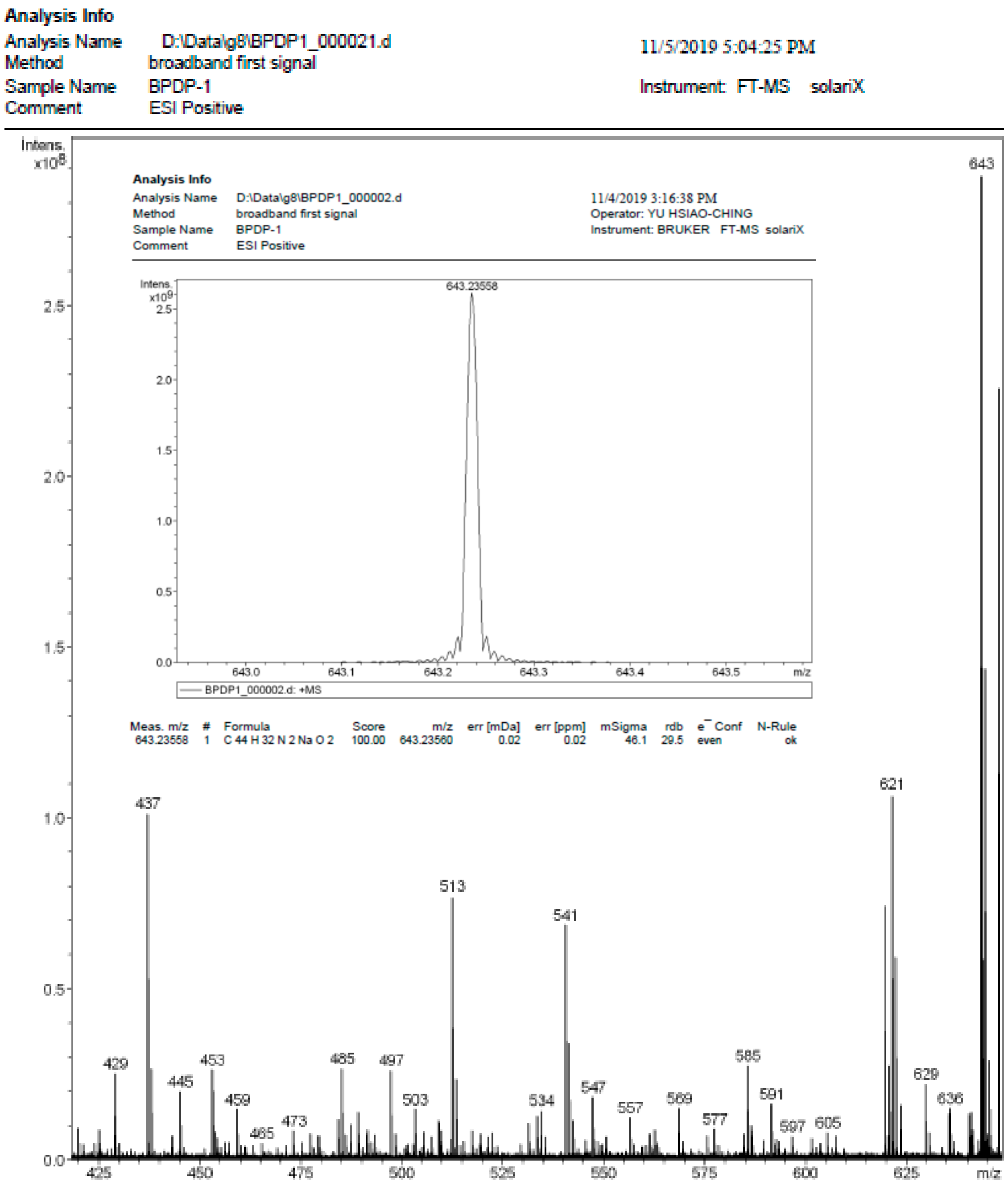

Figure S6. Mass and HR-MS of the **BPDP-D**.
